# A real-world pharmacovigilance study of polatuzumab vedotin based on the FDA adverse event reporting system (FAERS)

**DOI:** 10.3389/fphar.2024.1405023

**Published:** 2024-06-25

**Authors:** Dan Liu, Wei Mao, Bin Hu, Xingxing Li, Quanfeng Zhao, Lin Zhang, Jing Hu

**Affiliations:** ^1^ Department of Pharmacy, The First Affiliated Hospital of Army Medical University (Third Military Medical University), Chongqing, China; ^2^ Department of Pharmacy, Nanan People’s Hospital of Chongqing, Chongqing, China

**Keywords:** polatuzumab vedotin, FAERS, pharmacovigilance, adverse events, antibody-drug conjugate

## Abstract

**Background:**

Polatuzumab vedotin, the first FDA-approved antibody-drug conjugate (ADC) targeting CD79b, is utilized in the treatment of previously untreated diffuse large B-cell lymphoma (DLBCL) or high-grade B-cell lymphoma (HGBL), as well as relapsed or refractory (R/R) DLBCL. Despite its approval, concerns persist regarding the long-term safety profile of polatuzumab vedotin. This study aims to evaluate the adverse events (AEs) associated with polatuzumab vedotin since its approval in 2019, utilizing data mining strategies applied to the FDA Adverse Event Reporting System (FAERS).

**Methods:**

Signal detection employed four methodologies, including reporting odds ratio (ROR), proportional reporting ratio (PRR), bayesian confidence propagation neural network (BCPNN), and multi-item gamma poisson shrinker (MGPS), to evaluate and quantify the signals of polatuzumab vedotin-associated AEs. Additionally, subgroup analyses based on patients age, gender, and fatal cases were conducted to investigate AEs occurrences in specific subpopulations.

**Results:**

A total of 1,521 reports listing polatuzumab vedotin as a “principal suspect (PS)” drug were collected from the FAERS database. Through concurrent compliance with four algorithms, 19 significant Standardized MedDRA Query (SMQ) AEs and 92 significant Preferred Term (PT) AEs were detected. Subgroup analyses revealed a higher incidence of PTs in male patients compared to female patients, increased likelihood of polatuzumab vedotin-associated AEs in elder patients (>65 years), and AEs with a high risk of fatal cases include: blood lactate dehydrogenase increased, cytopenia, and hydronephrosis. The median time to AEs occurrence following polatuzumab vedotin initiation was 18.5 (5∼57.75) days, with 95% of AEs occurred within 162 days.

**Conclusion:**

This study identified various AEs associated with polatuzumab vedotin, offering critical insights for clinical monitoring and risk identification in patients receiving polatuzumab vedotin therapy.

## Introduction

Diffuse large B-cell lymphoma (DLBCL) is a heterogeneous and invasive tumor originating from mature B-cells, comprising 35%∼40% of all non-Hodgkin’s lymphoma (Testoni et al., 2015). Its incidence is on the rise annually (Cultrera and Dalia, 2012; Goy et al., 2019). Approximately 60% of DLBCL patients have received standard chemotherapy, however, 30%–40% of patients may encounter recurrence or progression to refractory DLBCL (Vodicka et al., 2022). Therefore, there is a critical need to explore novel drugs and treatment for recurrent or refractory DLBCL, with expanding therapeutic prospects through targeted therapy development. Some approved second-line or higher treatments include the combination therapy of polatuzumab vedotin, an antibody drug conjugate, in combination with Bendamustine (B) and Rituximab (R) (Sehn et al., 2022), and the combination of anti-CD19 monoclonal antibody tafasitamab with lenalidomide (Belada et al., 2023).

Polatuzumab vedotin is a novel humanized anti-CD79b antibody coupled with the anti-mitotic agent monomethylastatin E (MMAE) to form an antibody-drug conjugate (ADC). It was rapidly approved by the US Food and Drug Administration (FDA) on 10 June 2019, in combination with BR for adult patients with relapsed/refractory (R/R) DLBCL who received at least two prior treatments (Deeks, 2019). On 19 April 2023, the FDA extended its approval for adult patients with previously untreated DLBCL or high-grade B-cell lymphoma (HGBL) (https://www.fda.gov/drugs/resources-information-approved-drugs/fda-approves-polatuzumab-vedotin-piiq-previously-untreated-diffuse-large-b-cell-lymphoma-not). Polatuzumab vedotin combines advantages of immunotherapy and chemotherapy, with its target CD79b showing over 96% positivity in B-cell tumor cells and minimal expression in normal cells, enhancing targeting precision and reducing side-effects (Polson et al., 2007; Dornan et al., 2009). Additionally, the conjugated anti-microtubule agent MMAE exhibits potent cytotoxicity against lymphoma cells with higher selectivity and robust targeting, expanding its clinical application prospects.

With the widespread use of polatuzumab vedotin concerns about its safety have risen in recent years. The previous phase II and III clinical studies have identified the most common adverse events (AEs), including neutropenia, thrombocytopenia, anemia, hair loss, peripheral neuropathy, fatigue, diarrhea, fever, loss of appetite, and pneumonia (Terui et al., 2021; Sehn et al., 2022; Song et al., 2023). However, long-term safety and efficacy data primarily rely on case reports or clinical trials. It is necessary to perform comprehensive real-world safety assessments owing to limitations in sample sizes, selection criteria, follow-up duration, and AEs focused on specific systems. Given the widespread clinical application of polatuzumab vedotin and the lack of real-world AEs assessment, this pharmacovigilance analysis is essential in evaluating its safety and promoting rational prescription.

The FDA Adverse Event Reporting System (FAERS), a public pharmacovigilance database, facilitates post-market safety monitoring of drugs and therapeutic products (Vogel et al., 2020; Setyawan et al., 2021). This study aims to evaluate the safety of polatuzumab vedotin by analyzing post-marketing FAERS data to provide a comprehensive and valuable reference for its rational clinical application.

## Materials and methods

### Data sources and procedures

The FAERS database, a public database, is used by the FDA to gather all spontaneously reported AE reports. Data from the launch of polatuzumab vedotin (10 June 2019) to the third quarter of 2023 were downloaded from the FAERS database and imported into PostgreSQL for management. In this study, the basic information (including age, sex and weight), reporting country, type of reporter, indications, suspicious drugs, AEs, time of onset of AEs, date of occurrence of AEs and outcome of AEs were extracted for analysis.

In this study, “polatuzumab vedotin” and the trade name “POLIVY” were used to identify the related records of polatuzumab vedotin. To enhance accuracy, only cases in which the drug has the effect of “primary suspect” (PS) in AEs and the patients aged 18 or above were used. The preferred term (PT) in MedDRA terminology was used to encode every report in the FAERS database. We used PT to identify AEs. In addition, we also grouped AEs into the Standardized MedDRA Query (SMQ)to describe disease conditions.

### Data mining algorithm

Based on a fourfold table, the reporting odds ratio (ROR), the proportional reporting ratio (PRR), the Bayesian confidence propagation neural network (BCPNN), and the multi-item gamma Poisson shrinker (MGPS) were applied to detect an association between various polatuzumab vedotin regimens and AEs in accordance with the disproportionality analysis. The equations and criteria for the above four algorithms are shown in Supplementary Table S1.

### The time to onset of AEs

Time to onset of AEs is calculated by subtracting the date of initiation of polatuzumab vedotin from the date of onset of AEs in the report. This study analyzed the time to AEs after polatuzumab vedotin use. The median and interquartile range were used to characterize the time to AEs, to compare the distribution of AEs by gender and age and the cumulative incidence of AEs.

### Subgroup analysis

According to the patient’s age (18–65 years old, >65 years old), gender (male and female), and death cases, the subgroup analysis was conducted to investigate the occurrence of AEs in the subgroup.

### Statistical analysis

The results were analyzed using descriptive statistics. Display the frequency (percentage) of categorical variables and continuous variables’ median (interquartile range). All data in this study were processed and analyzed using PostgreSQL (version 15.3), R software (version 4.3.1), and MedDRA (version 26.1).

## Results

### Descriptive analysis

From 10 June 2019, to the third quarter of 2023, FAERS received 1,521 reports of AEs using polatuzumab vedotin. The median age of patients in the reports was 73 years old, with a median weight of 59.8 kg, male patients were the majority (49.0%). 99.6% were urgent reports, and medical authors reported 95.4%. The reporting countries are mainly Japan (45.3%), the United States (9.4%), and Germany (4.7%). The patient’s diagnosis is mainly diffuse large B-cell lymphoma (78.9%). The main accompanying medications are Rituximab (71.1%), Bendamustine (54.4%), and Cyclophosphamide (23.7%). The reported AE outcomes were mostly death (28.7%) and prolonged hospitalization (25.5%), as indicated in Table 1.

**TABLE 1:** Clinical characteristics of reports with polatuzumab vedotin from the FAERS database.

Characteristic	Overall
	(*N* = 1,521)
Age	
Median (Q1, Q3)	73.0 (65.0, 80.0)
>65	829 (54.5%)
18∼65	293 (19.3%)
Missing	399 (26.2%)
Gender	
Female	552 (36.3%)
Male	745 (49.0%)
Missing	224 (14.7%)
Weight	
Median (Q1, Q3)	59.8 (50.2, 72.2)
Missing	866 (56.9%)
The type of report submitted	
Direct	6 (0.4%)
Expedited	1,515 (99.6%)
Occupation of the reporter	
Physician	1,285 (84.5%)
Pharmacist	91 (6.0%)
Other health-professional	74 (4.9%)
Consumer	67 (4.4%)
Missing	4 (0.3%)
Outcome of patient	
Death	437 (28.7%)
Life-threatening	27 (1.8%)
Hospitalization-initial or prolonged	388 (25.5%)
Disability	18 (1.2%)
Date FDA received case	
2019 (2019/6/10∼2019/12/31)	54 (3.6%)
2020	135 (8.9%)
2021	220 (14.5%)
2022	658 (43.3%)
2023 (2023/1/1∼2023/9/30)	454 (29.8%)
Country of the reporter (Top 5)	
Japan	689 (45.3%)
United States	143 (9.4%)
Germany	72 (4.7%)
United Kingdom	54 (3.6%)
Poland	39 (2.6%)
Indications (Top 5)	
Diffuse large B-cell lymphoma	1,090 (71.7%)
Diffuse large B-cell lymphoma refractory	80 (5.3%)
Diffuse large B-cell lymphoma recurrent	30 (2.0%)
B-cell lymphoma	25 (1.6%)
Follicular lymphoma	20 (1.3%)

Note: “Missing” indicates that the value is missing.

### SMQ level signal detection

As demonstrated in Table 2, a total of 19 polatuzumab vedotin-related SMQ level AEs were detected after completing four algorithms simultaneously. It can be seen that the signal of myelodysplastic syndrome is the strongest (ROR = 12.81, PRR = 11.82, IC = 3.50, EBGM = 11.79), followed by agranulocytosis (ROR = 12.69, PRR = 9.64, IC = 3.25, EBGM = 9.62) and hematopoietic leukopenia (ROR = 11.87, PRR = 9.49, IC = 3.23, EBGM = 9.48). Other serious AEs include haematopoietic thrombocytopenia (ROR = 11.41, PRR = 10.27, IC = 3.32, EBGM = 10.25), haematopoietic cytopenias affecting more than one type of blood cell (ROR = 10.35, PRR = 9.47, IC = 3.20, EBGM = 9.46), haematopoietic erythropenia (ROR = 5.75, PRR = 5.39, IC = 2.40, EBGM = 5.39), and sepsis (ROR = 5.57, PRR = 5.28, IC = 2.37, EBGM = 5.28). Other rare signals include biliary system related investigations, signs and symptoms, liver related investigations, signs and symptoms, drug reaction with eosinophilia and systemic symptoms syndrome, COVID-19, tumor lysis syndrome, infective pneumonia, etc.

**TABLE 2:** Signal strength of reports of polatuzumab vedotin at the SMQ level in FAERS database.

SMQ	Case number	ROR (95% CI)	PRR (χ^2^)	IC (IC_025_)	EBGM (EBGM_05_)
Myelodysplastic syndrome	127	12.81 (10.68∼15.37)	11.82 (1,263.94)	3.50 (3.21)	11.79 (10.13)
Agranulocytosis	397	12.69 (11.32∼14.23)	9.64 (3,153.71)	3.25 (3.09)	9.62 (8.74)
Haematopoietic leukopenia	332	11.87 (10.51∼13.40)	9.49 (2,576.78)	3.23 (3.04)	9.48 (8.56)
Haematopoietic thrombocytopenia	167	11.41 (9.72∼13.41)	10.27 (1,409.03)	3.32 (3.06)	10.25 (8.96)
Haematopoietic cytopenias affecting more than one type of blood cell	142	10.35 (8.70∼12.30)	9.47 (1,084.69)	3.20 (2.92)	9.46 (8.18)
Haematopoietic erythropenia	115	5.75 (4.75∼6.95)	5.39 (416.61)	2.40 (2.09)	5.39 (4.59)
Sepsis	96	5.57 (4.53∼6.85)	5.28 (336.72)	2.37 (2.03)	5.28 (4.44)
Liver infections	11	4.94 (2.73∼8.95)	4.91 (34.29)	2.07 (1.04)	4.91 (2.99)
Infectious biliary disorders	9	4.78 (2.48∼9.21)	4.76 (26.74)	1.99 (0.85)	4.76 (2.75)
Systemic lupus erythematosus	256	4.60 (4.03∼5.27)	4.00 (600.21)	1.99 (1.78)	3.99 (3.57)
Opportunistic infections	338	4.52 (4.01∼5.11)	3.74 (721.12)	1.90 (1.72)	3.74 (3.38)
Gastrointestinal perforation	25	4.28 (2.89∼6.36)	4.23 (61.85)	1.99 (1.32)	4.23 (3.04)
Gastrointestinal obstruction	28	4.04 (2.78∼5.87)	3.98 (62.79)	1.92 (1.29)	3.98 (2.91)
Biliary system related investigations, signs and symptoms	20	3.93 (2.53∼6.11)	3.89 (43.05)	1.86 (1.11)	3.89 (2.69)
Liver related investigations, signs and symptoms	73	3.38 (2.67∼4.27)	3.26 (116.11)	1.68 (1.29)	3.26 (2.68)
Drug reaction with eosinophilia and systemic symptoms syndrome	669	3.31 (2.99∼3.66)	2.30 (604.50)	1.20 (1.07)	2.29 (2.11)
COVID-19	112	3.19 (2.63∼3.87)	3.03 (156.10)	1.59 (1.27)	3.03 (2.58)
Tumour lysis syndrome	118	2.95 (2.45∼3.56)	2.80 (140.34)	1.47 (1.17)	2.80 (2.39)
Infective pneumonia	216	2.54 (2.20∼2.93)	2.32 (172.83)	1.21 (0.98)	2.32 (2.06)

Abbreviations: SMQ, Standardized MedDRA queries; ROR, reporting odds ratio; CI, confidence interval; PRR, proportional reporting ratio; 95% CI, 95% confidence interval; χ^2^, chi-squared; IC, information component; IC_025_, the lower limit of the 95% CI of the IC; EBGM, empirical Bayesian geometric mean; EBGM_05_, empirical Bayesian geometric mean lower 95% CI for the posterior distribution.

### PT level signal detection

After complying with four algorithms simultaneously, 92 PT level AEs related to polatuzumab vedotin were detected, as shown in Table 3. The results revealed that 15 systems were involved in the AE signals associated with polatuzumab vedotin. The top five systemic organs (SOC) reported by polatuzumab vedotin-related AEs were blood and lymphatic system disorders (503 cases), investigations (502 cases), infections and infestations (394 cases), metabolism and nutrition disorders (106 cases), general disorders and administration site conditions (99 cases). Among them, blood and lymphatic system diseases involve 15 PT signals, mainly including anemia (102 cases), febrile neutrtropenia (86 cases), neutropenia (81 cases), thrombocytopenia (57 cases), and so on. Among the top 20 ranked PTs in the number of reports, neutrophil count decreased, anaemia, platelet count decreased, white blood cell count decreased, febrile neutropenia, pyrexia, neutropenia, thrombocytopenia, lymphocyte count decreased, pneumonia, decreased appetite, cytopenia, myelosuppression, cytomegalovirus infection, neuropathy peripheral, tumour lysis syndrome, sepsis and infection are AEs included in the polatuzumab vedotin manual; COVID-19 and blood lactate dehydrogenase increased is not explicitly indicated in the polatuzumab vedotin manual and deserves further evaluation.

**TABLE 3:** Signal strength of reports of polatuzumab vedotin at the PT level in FAERS database.

System organ class	Preferred terms	Case number	ROR (95% CI)	PRR (χ^2^)	IC (IC_025_)	EBGM (EBGM_05_)
Blood and lymphatic system disorders	Abdominal lymphadenopathy	4	47.82 (17.83∼128.26)	47.70 (180.89)	2.94 (1.18)	47.19 (20.67)
	Hypofibrinogenaemia	3	40.39 (12.94∼126.04)	40.31 (113.96)	2.61 (0.54)	39.95 (15.42)
	Cytopenia	45	38.46 (28.55∼51.80)	37.35 (1,579.76)	4.73 (4.23)	37.04 (28.87)
	Febrile neutropenia	86	18.26 (14.68∼22.70)	17.28 (1,318.12)	3.98 (3.62)	17.22 (14.34)
	Myelosuppression	45	15.61 (11.60∼21.01)	15.18 (595.09)	3.71 (3.22)	15.13 (11.80)
	Disseminated intravascular coagulation	7	10.21 (4.86∼21.47)	10.17 (57.76)	2.66 (1.35)	10.15 (5.45)
	Haematotoxicity	7	9.97 (4.74∼20.96)	9.93 (56.08)	2.64 (1.33)	9.91 (5.32)
	Anaemia	102	8.87 (7.26∼10.85)	8.35 (663.52)	3.01 (2.68)	8.33 (7.04)
	Thrombocytopenia	57	7.80 (5.98∼10.16)	7.54 (324.66)	2.83 (2.39)	7.53 (6.04)
	Neutropenia	81	7.48 (5.98∼9.36)	7.14 (429.97)	2.78 (2.41)	7.13 (5.91)
	Pancytopenia	24	6.85 (4.58∼10.26)	6.76 (117.92)	2.60 (1.91)	6.75 (4.82)
	Leukopenia	19	5.74 (3.65∼9.03)	5.68 (73.42)	2.34 (1.57)	5.68 (3.89)
	Lymphopenia	6	5.11 (2.29∼11.39)	5.09 (19.71)	1.95 (0.54)	5.08 (2.60)
	Agranulocytosis	6	4.85 (2.17∼10.82)	4.83 (18.24)	1.90 (0.48)	4.83 (2.47)
	Lymphadenopathy	11	4.59 (2.53∼8.30)	4.56 (30.60)	1.98 (0.96)	4.56 (2.77)
Cardiac disorders	Cardiac failure	19	3.47 (2.21∼5.45)	3.44 (32.92)	1.69 (0.92)	3.43 (2.35)
Gastrointestinal disorders	Gastrointestinal perforation	10	40.71 (21.80∼76.04)	40.45 (381.27)	3.81 (2.73)	40.09 (23.77)
	Subileus	3	26.73 (8.58∼83.27)	26.68 (73.71)	2.51 (0.44)	26.53 (10.25)
	Ileus	13	20.25 (11.72∼35.00)	20.09 (234.80)	3.55 (2.61)	20.00 (12.65)
	Enterocolitis	5	11.42 (4.74∼27.51)	11.39 (47.26)	2.55 (0.99)	11.36 (5.44)
	Intestinal perforation	6	8.78 (3.94∼19.59)	8.75 (41.13)	2.45 (1.04)	8.74 (4.46)
	Upper gastrointestinal haemorrhage	6	4.96 (2.22∼11.07)	4.95 (18.88)	1.92 (0.51)	4.94 (2.53)
	Colitis	11	3.85 (2.13∼6.97)	3.83 (23.05)	1.77 (0.75)	3.83 (2.33)
	Gastrointestinal haemorrhage	15	3.31 (1.99∼5.51)	3.29 (23.95)	1.61 (0.74)	3.29 (2.15)
General disorders and administration site conditions	Mucosal inflammation	11	6.36 (3.51∼11.51)	6.32 (49.23)	2.36 (1.33)	6.31 (3.84)
	Decreased activity	5	5.40 (2.24∼13.00)	5.39 (17.85)	1.94 (0.38)	5.38 (2.58)
	Pyrexia	83	3.64 (2.92∼4.55)	3.50 (150.36)	1.78 (1.42)	3.50 (2.91)
Hepatobiliary disorders	Cholecystitis	5	8.37 (3.47∼20.15)	8.34 (32.27)	2.32 (0.76)	8.33 (3.99)
	Hepatic function abnormal	20	8.00 (5.14∼12.44)	7.91 (120.67)	2.76 (2.01)	7.90 (5.46)
	Hyperbilirubinaemia	4	6.17 (2.31∼16.47)	6.16 (17.26)	1.97 (0.20)	6.15 (2.70)
	Hepatotoxicity	7	3.84 (1.83∼8.07)	3.82 (14.61)	1.69 (0.38)	3.82 (2.05)
Immune system disorders	Hypogammaglobulinaemia	18	35.76 (22.43∼57.03)	35.35 (596.19)	4.19 (3.40)	35.07 (23.74)
	Cytokine release syndrome	21	12.62 (8.20∼19.43)	12.46 (220.97)	3.30 (2.56)	12.43 (8.66)
	Haemophagocytic lymphohistiocytosis	4	4.88 (1.83∼13.02)	4.87 (12.28)	1.77 (0.00)	4.86 (2.14)
Infections and infestations	Cytomegalovirus enterocolitis	8	148.53 (73.28∼301.05)	147.76 (1,127.77)	3.93 (2.72)	142.93 (79.14)
	Cytomegalovirus hepatitis	3	124.25 (39.39∼391.93)	124.01 (355.90)	2.74 (0.67)	120.59 (46.12)
	Cytomegalovirus infection reactivation	17	35.59 (22.02∼57.51)	35.20 (560.58)	4.15 (3.33)	34.93 (23.38)
	Cytomegalovirus infection	38	30.96 (22.41∼42.77)	30.21 (1,066.84)	4.45 (3.91)	30.01 (22.90)
	Pneumonia cytomegaloviral	4	29.01 (10.84∼77.65)	28.94 (107.18)	2.82 (1.05)	28.75 (12.61)
	Fungaemia	3	25.19 (8.09∼78.43)	25.14 (69.14)	2.50 (0.43)	25.00 (9.66)
	Cytomegalovirus chorioretinitis	4	25.04 (9.36∼67.00)	24.98 (91.57)	2.77 (1.00)	24.84 (10.90)
	Cytomegalovirus viraemia	9	22.72 (11.78∼43.83)	22.59 (184.82)	3.40 (2.26)	22.48 (12.97)
	Staphylococcal sepsis	4	15.88 (5.94∼42.44)	15.84 (55.42)	2.58 (0.81)	15.79 (6.94)
	Coronavirus infection	19	15.55 (9.88∼24.46)	15.37 (254.49)	3.49 (2.72)	15.31 (10.48)
	Bacterial sepsis	3	14.94 (4.80∼46.47)	14.92 (38.82)	2.32 (0.25)	14.87 (5.75)
	Oesophageal candidiasis	3	12.07 (3.88∼37.52)	12.05 (30.31)	2.22 (0.15)	12.01 (4.65)
	Bacteraemia	10	11.64 (6.25∼21.70)	11.57 (96.38)	2.94 (1.86)	11.54 (6.86)
	Progressive multifocal leukoencephalopathy	6	10.87 (4.87∼24.26)	10.83 (53.44)	2.62 (1.21)	10.81 (5.52)
	Hepatitis b reactivation	4	10.86 (4.06∼29.00)	10.83 (35.61)	2.37 (0.61)	10.81 (4.75)
	Pneumocystis jirovecii pneumonia	8	9.17 (4.58∼18.39)	9.13 (57.83)	2.63 (1.41)	9.11 (5.09)
	Escherichia infection	4	6.80 (2.55∼18.16)	6.79 (19.71)	2.05 (0.28)	6.78 (2.98)
	Vascular device infection	4	6.75 (2.53∼18.01)	6.73 (19.50)	2.04 (0.27)	6.72 (2.96)
	Clostridium difficile colitis	4	5.86 (2.19∼15.63)	5.84 (16.04)	1.92 (0.16)	5.84 (2.57)
	Sepsis	38	5.26 (3.81∼7.27)	5.16 (127.85)	2.29 (1.75)	5.15 (3.94)
	Staphylococcal infection	9	4.85 (2.52∼9.34)	4.83 (27.31)	2.01 (0.87)	4.82 (2.79)
	Septic shock	14	4.82 (2.85∼8.17)	4.79 (42.00)	2.08 (1.18)	4.78 (3.08)
	COVID-19 pneumonia	10	4.75 (2.55∼8.84)	4.72 (29.34)	2.00 (0.93)	4.72 (2.80)
	Infection	36	3.31 (2.37∼4.60)	3.25 (56.48)	1.66 (1.10)	3.25 (2.46)
	COVID-19	76	2.64 (2.09∼3.32)	2.55 (73.25)	1.34 (0.96)	2.55 (2.10)
	Pneumonia	56	2.57 (1.96∼3.35)	2.51 (51.48)	1.31 (0.86)	2.51 (2.00)
Injury, poisoning and procedural complications	Femoral neck fracture	4	12.38 (4.63∼33.07)	12.35 (41.61)	2.45 (0.68)	12.32 (5.41)
	Infusion related reaction	19	3.26 (2.07∼5.13)	3.23 (29.39)	1.61 (0.84)	3.23 (2.21)
Investigations	Cytomegalovirus test positive	21	187.46 (120.77∼290.97)	184.89 (3,684.07)	5.12 (4.39)	177.37 (122.78)
	Blood lactate dehydrogenase increased	31	39.16 (27.39∼55.97)	38.38 (1,119.23)	4.58 (3.98)	38.05 (28.22)
	Neutrophil count decreased	116	38.95 (32.21∼47.11)	36.06 (3,929.82)	4.96 (4.65)	35.77 (30.51)
	Lymphocyte count decreased	57	34.85 (26.72∼45.45)	33.58 (1790.08)	4.70 (4.26)	33.33 (26.69)
	Platelet count decreased	111	14.82 (12.21∼17.98)	13.81 (1,321.80)	3.70 (3.39)	13.77 (11.71)
	Amylase increased	3	12.02 (3.87∼37.38)	12.00 (30.17)	2.22 (0.15)	11.97 (4.63)
	Blood alkaline phosphatase increased	11	9.77 (5.39∼17.68)	9.70 (85.73)	2.81 (1.79)	9.68 (5.89)
	White blood cell count decreased	80	9.60 (7.66∼12.03)	9.15 (582.88)	3.12 (2.75)	9.13 (7.56)
	Gamma-glutamyltransferase increased	9	7.86 (4.08∼15.14)	7.82 (53.44)	2.52 (1.38)	7.80 (4.51)
	Aspartate aminotransferase increased	20	7.04 (4.53∼10.95)	6.96 (102.18)	2.60 (1.85)	6.95 (4.81)
	Alanine aminotransferase increased	20	5.72 (3.68∼8.89)	5.66 (76.72)	2.34 (1.59)	5.65 (3.90)
	C-reactive protein increased	17	5.54 (3.43∼8.93)	5.49 (62.42)	2.28 (1.46)	5.48 (3.67)
	Blood bilirubin increased	6	4.26 (1.91∼9.50)	4.25 (14.90)	1.76 (0.35)	4.24 (2.17)
Metabolism and nutrition disorders	Tumour lysis syndrome	28	39.97 (27.46∼58.19)	39.25 (1,034.97)	4.55 (3.92)	38.91 (28.42)
	Hypoalbuminaemia	7	14.49 (6.89∼30.48)	14.43 (87.21)	2.93 (1.62)	14.38 (7.72)
	Hyperuricaemia	3	11.34 (3.65∼35.26)	11.32 (28.16)	2.19 (0.12)	11.30 (4.37)
	Hypercalcaemia	5	5.76 (2.39∼13.88)	5.75 (19.60)	2.00 (0.44)	5.74 (2.75)
	Hypokalaemia	17	5.60 (3.47∼9.04)	5.55 (63.44)	2.29 (1.48)	5.54 (3.71)
	Decreased appetite	46	2.82 (2.10∼3.78)	2.76 (52.19)	1.44 (0.95)	2.76 (2.16)
Nervous system disorders	Peripheral sensory neuropathy	10	26.54 (14.22∼49.52)	26.37 (242.66)	3.57 (2.50)	26.22 (15.56)
	Immune effector cell-associated neurotoxicity syndrome	5	11.49 (4.77∼27.67)	11.45 (47.59)	2.55 (0.99)	11.42 (5.47)
	Polyneuropathy	8	8.28 (4.13∼16.60)	8.24 (50.84)	2.53 (1.32)	8.23 (4.60)
	Neuropathy peripheral	44	6.07 (4.49∼8.19)	5.92 (180.59)	2.49 (1.99)	5.91 (4.60)
Renal and urinary disorders	Azotaemia	3	14.58 (4.69∼45.33)	14.55 (37.73)	2.31 (0.24)	14.50 (5.61)
	Hydronephrosis	6	11.78 (5.28∼26.30)	11.74 (58.82)	2.68 (1.27)	11.71 (5.98)
	Cystitis haemorrhagic	3	11.38 (3.66∼35.39)	11.36 (28.28)	2.19 (0.13)	11.34 (4.39)
Respiratory, thoracic and mediastinal disorders	Organising pneumonia	5	13.73 (5.70∼33.07)	13.68 (58.61)	2.67 (1.10)	13.64 (6.54)
	Interstitial lung disease	13	3.72 (2.15∼6.42)	3.69 (25.58)	1.75 (0.81)	3.69 (2.34)
Skin and subcutaneous tissue disorders	Erythema multiforme	4	7.97 (2.98∼21.28)	7.95 (24.26)	2.16 (0.40)	7.94 (3.49)
Vascular disorders	Thrombophlebitis	4	23.08 (8.63∼61.75)	23.03 (83.84)	2.74 (0.97)	22.91 (10.06)

Abbreviations: PT, preferred term; ROR, reporting odds ratio; CI, confidence interval; PRR, proportional reporting ratio; 95% CI, 95% confidence interval; χ^2^, chi-squared; IC, information component; IC_025_, the lower limit of the 95% CI of the IC; EBGM, empirical Bayesian geometric mean; EBGM_05_, empirical Bayesian geometric mean lower 95% CI for the posterior distribution.

### Subgroup analysis

**FIGURE 1:**
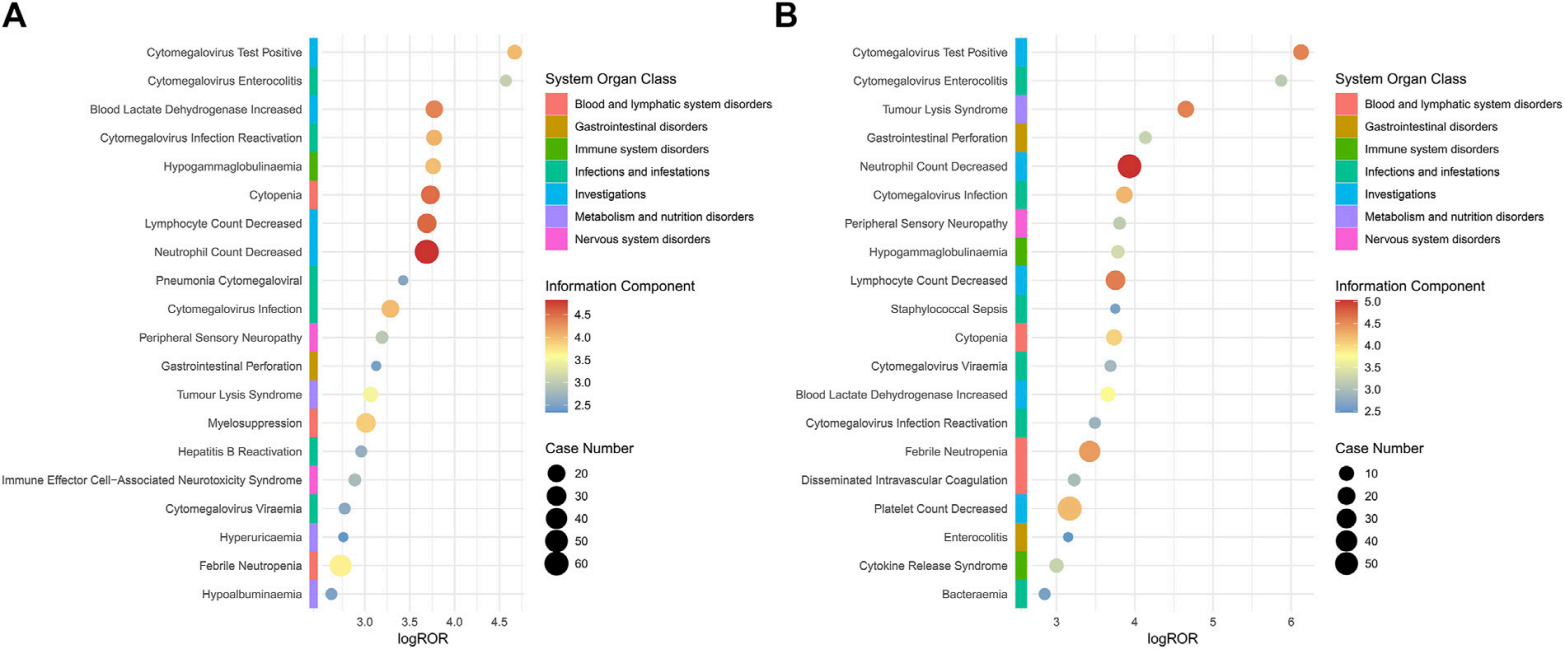
Subgroup analysis based on gender for polatuzumab vedotin related AEs. **(A)** Male group; **(B)** Female group. Abbreviations: ROR, reporting odds ratio; log ROR, logarithm of the reporting ROR; AEs, adverse events.

This study conducted a subgroup analysis of AEs caused by the use of polatuzumab vedotin in different genders and age groups. Additionally, an analysis was conducted on cases of AEs that resulted in death after taking polatuzumab vedotin. The results indicated that the order of signal intensity was consistent for each of the four signal detection methods, which involved 54 PTs in male patients and 49 PTs in female patients, with male patients having a higher number of PTs than female patients (Supplementary Table S2). Among the top 10 related AEs with ROR signal intensity, male patients are mainly involved in AEs after using polatuzumab vedotin, including neutral count decreased, lymphocyte count decreased, cytopia, cytomegalovirus infection, and blood lactate dehydrogenase increased. The main AEs in female patients using polatuzumab vedotin include neutrophil count decreased, lymphocyte count decreased, tumour lysis syndrome, cytomegalovirus infection, and cytomegalovirus test positive. The details are shown in Figure 1.

**FIGURE 2:**
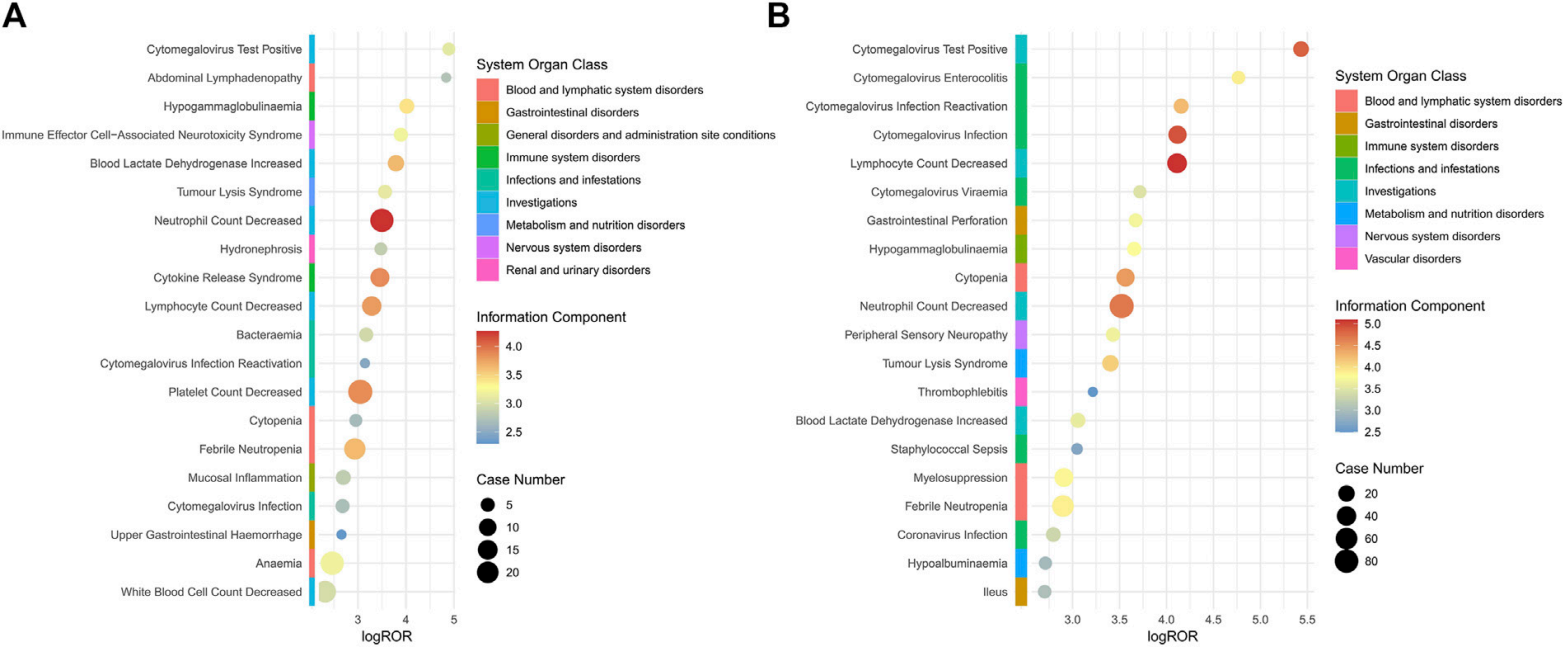
Subgroup analysis based on age for polatuzumab vedotin related AEs. **(A)** 18–65 years old (younger patients); **(B)** >65 years of old (elder patients). Abbreviations: ROR, reporting odds ratio; log ROR, logarithm of the reporting ROR; AEs, adverse events.

Age stratified analysis revealed that patients greater than 65 years of age were involved in 51 PTs and patients less than 65 years of age were involved in 36 PTs, and that individuals who were greater than 65 years of age were more likely to experience polatuzumab vedotin-related AEs, as depicted in Supplementary Table S3. The primary side effects of polatuzumab vedotin for patients over 65 years old include neutrophil count decreased, lymphocyte count decreased, cytomegalovirus infection, cytopenia, and cytomegalovirus test positive. The main AEs associated with the use of polatuzumab vedotin in patients under the age of 65 include neutral count decreased, lymphocyte count decreased, cytokine release syndrome, and blood lactate dehydrogenase increased. The details are shown in Figure 2.

**FIGURE 3:**
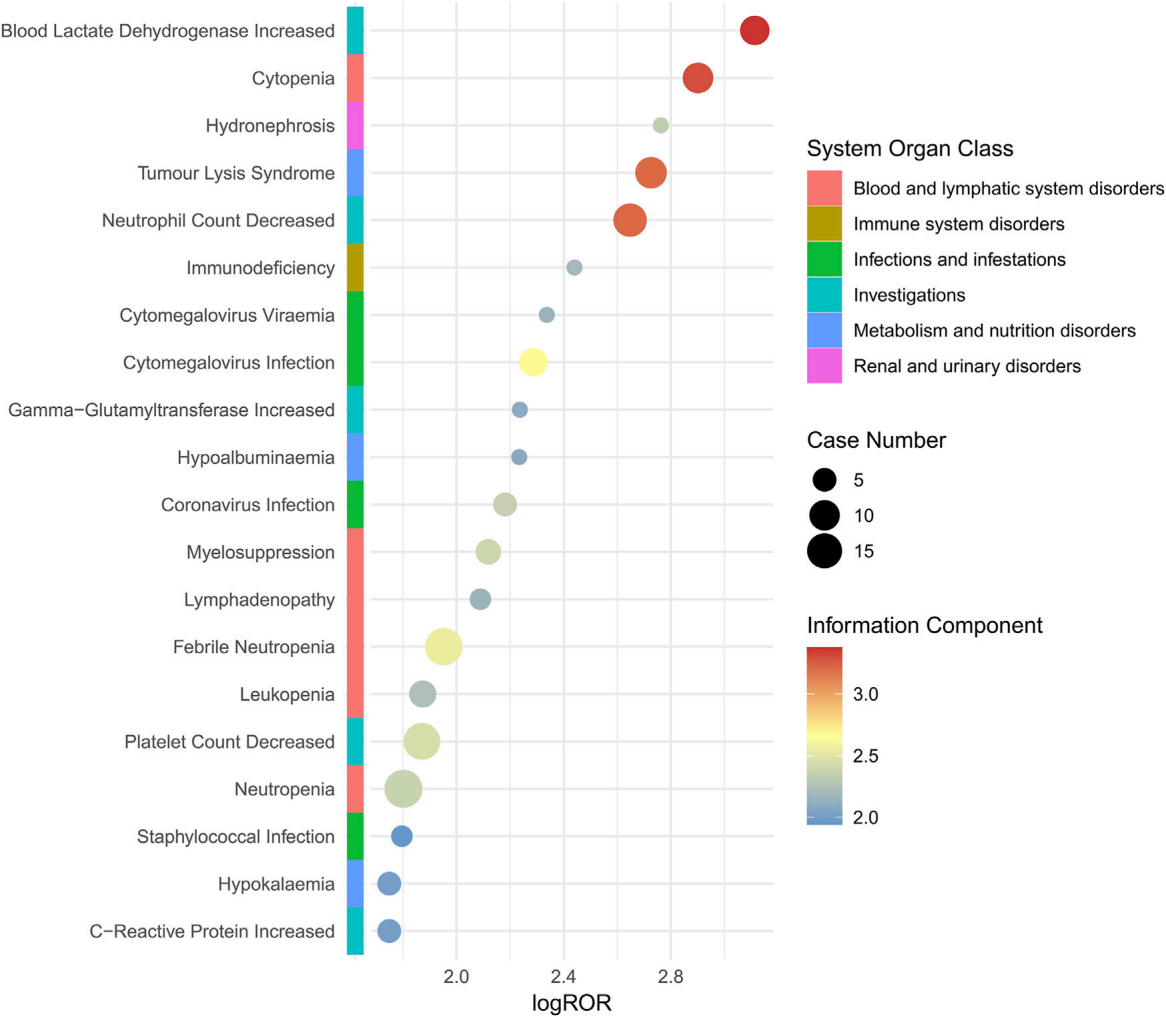
Subgroup analysis based on mortality cases for polatuzumab vedotin related AEs. Abbreviations: ROR, reporting odds ratio; log ROR, logarithm of the reporting ROR; AEs, adverse events.

A total of 28 PTs were involved in death cases, as shown in Supplementary Table S4. High-risk AEs in fatal cases include blood lactate dehydrogenase increased, cytopenia, and hydronephrosis. It is recommended to enhance monitoring of such AEs when utilizing this drug. The study demonstrated that blood lactate dehydrogenase increased, hydronephrosis and hypogammaglobulinaemia were manifestations of PT signaling that were not mentioned in the drug instructions, as illustrated in Figure 3.

### Analysis of time to polatuzumab vedotin-induced AEs onset

**FIGURE 4:**
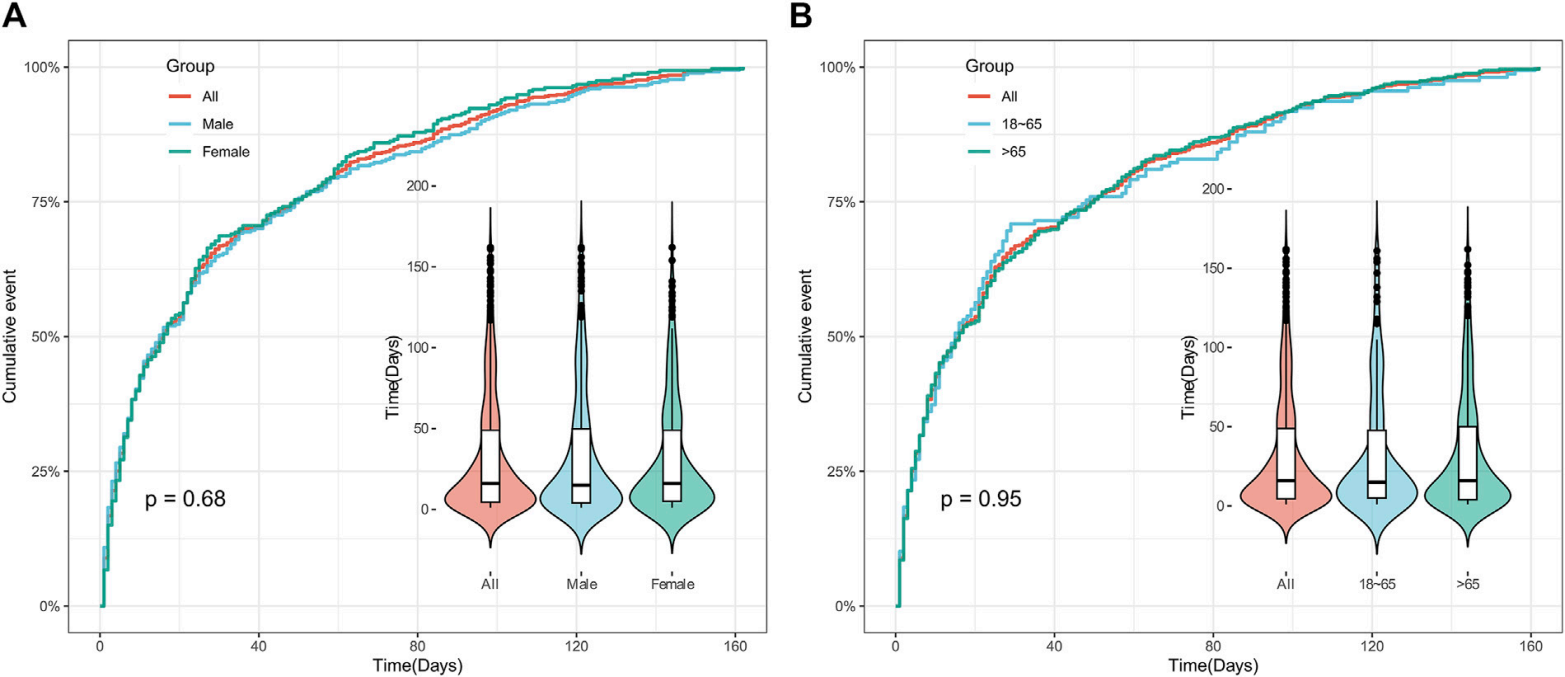
Time to onset of polatuzumab vedotin related AEs. **(A)** Accumulated incidence of AEs by gender within 162 days; **(B)** The cumulative incidence rate of AEs in different age groups within 162 days. Abbreviations: AEs, adverse events.

The time of AEs was calculated by subtracting the date of AE occurrence in the report from the date when polatuzumab vedotin was first used in this study. It was found that the median time for AEs was 18.5 (5–57.75) days after initiation of treatment with polatuzumab vedotin, and 95% of cases experienced AEs within 162 days. Meanwhile, we examined the distribution of AEs that happened within 162 days of taking polatuzumab vedotin based on gender and age, and there was no statistically significant difference. Additional information can be found in Supplementary Table S5 and Figure 4.

## Discussion

In previous studies, research on polatuzumab vedotin has primarily focused on mechanisms, clinical trials, and literature analysis, with limited real-world post-marketing studies. Therefore, we utilized the FAERS database to comprehensively investigate the post-marketing AEs reports associated with polatuzumab vedotin to provide a basis for rational clinical use.

Our study revealed a higher rated of AEs reporting in men compared to women, potentially attributed to an increased male population diagnosed with DLBCL, consequently leading to more medication opportunities. Furthermore, our study suggested a high proportion of AEs in elderly patients (>65 years old), likely due to the increased DLBCL incidence with age, especially among individuals over 65 years old (Morgan et al., 1997; Morton et al., 2006; Hounsome et al., 2022). Elderly patients are also at higher risk of severe infections and exhibit decreased tissue/organ tolerance to drugs with age, rendering them more susceptible to AEs (Wildiers and de Glas, 2020; Thandra et al., 2021). With the expanding clinical use of polatuzumab vedotin, clinicians need to remain vigilant regarding associated AEs, particularly in elderly patients. Healthcare professionals accounted for approximately 95.4% of AE reports, with reporting primarily concentrated in Japan, the United States, and Germany, reflecting the frequent use of polatuzumab vedotin in these countries. The use of polatuzumab vedotin may lead to severe adverse events (SAEs), with approximately 54% of reported cases resulting in hospitalization or prolonged hospital stays. Although not all AEs may be directly caused by polatuzumab vedotin treatment, special attention must be paid to those associated with polatuzumab vedotin, especially SAEs.

In this study, 92 signals involving 15 SOC were mined, including blood and lymphatic system disorders, metabolism and nutrition disorders, general disorders and administration site conditions, gastrointestinal disorders, nervous system disorders, immune system disorders, hepatobiliary disorders, cardiac disorders, respiratory, thoracic, and mediastinal disorders, renal and urinary disorders, skin and subcutaneous tissue disorders, vascular disorders, etc.

High-frequency and high-signal-intensity SOCs were primarily observed in reactions such as blood and lymphatic system disorders, investigations, infections, and infestations, consistent with findings from a global randomized phase III study (Song et al., 2023). The blood systems with a higher frequency of occurrence in these systems include anaemia, febrile neutropenia, and neutropenia; investigations include neutrophil count decreased, platelet count decreased, and white blood cell count decreased; COVID-19, pneumonia, and cytomegalovirus infection have a higher frequency of occurrence in infections and infestations. These AEs are commonly reported in clinical studies and instruction manuals (Phillips et al., 2022; Sehn et al., 2022). Infusion-related reactions were observed in approximately one-third of patients across various systems, with antihistamines and antipyretics recommended prior to treatment to mitigate these reactions. Despite preoperative medication, a small percentage of patients experienced low-grade infusion reactions (Assi et al., 2021). Anemia was the most common AE in the blood system, often accompanied by grade 3 to 4 AEs such as neutropenia and leukopenia, consistent with previous research (Terui et al., 2021; Song et al., 2023). Additionally, peripheral neuropathy, usually dose and duration-dependent, is a common AEs to polatuzumab vedotin, with dosage adjustments and supportive therapy recommended for control (Tilly et al., 2019). Infections and infestations, particularly pneumonia, fungal pneumonia, and sepsis, are the leading causes of fatal AEs, emphasizing the importance of vigilant medication monitoring during treatment (Sehn et al., 2020).

The top ten signals identified in this study, including blood alkaline phosphatase incremented, intrinsic performance, cholecystitis, and erythema multiform, were not explicitly mentioned in the polatuzumab vedotin instruction manual. High attention should be given to these emerging signals. Pneumocystis jirovecii pneumonia, polyeuropathy, hepatic function abnormal, haematotoxicity may be associated with the unspecific distribution of polatuzumab vedotin in multiple hyperperfusion organs, including the lungs, heart, liver, spleen, and kidneys, where ADC is used to decompose MMAE and other metabolites that contain MMAE (Yip et al., 2021). However, further research is necessary to determine the causal relationship between AEs and drugs. Anaemia, white blood cell count decreased, is one of the most common AEs of polatuzumab vedotin. Especially, anemia often leads to patient discontinuation or reduction of dosage (Terui et al., 2021).

The most common grade 3 AEs are those related to the blood and lymphatic system among DLBCL patients who receive polatuzumab vedotin treatment. The role of unbound MMAE in the cycle could be the cause of this effect (Camus and Tilly, 2021). Hematotoxicity is one of the most common dose-limiting adverse reactions in cancer treatment. Routine chemotherapy can be used to target blood cells that renew quickly, and this toxicity can result in a range of blood diseases (Haglund et al., 2010). Blood toxicity is a frequent AE of polatuzumab vedotin, and it includes anemia, thrombocytopenia, and neutropenia, all of which have been noted in the drug instructions. Our research results confirm this precisely. One of the most common and severe hematotoxic effects is anemia, which can cause polatuzumab vedotin treatment to be stopped and interfere with its effectiveness (Terui et al., 2021). Recombinant human erythropoietin (rhuEPO) is thought to be an effective method for treating anemia in cancer patients who undergo chemotherapy (Ozsahin et al., 2005). The majority of polatuzumab vedotin-induced neutropenia is characterized by laboratory abnormalities. In most cases, neutropenia is temporary, reversible, and does not result in treatment interruption. According to the published guidelines (Smith et al., 2006), restoring neutrophil count and continuing treatment can be achieved by administering growth factors and increasing the cycle duration to 28 days. Most clinical studies have reported neutropenia, mainly grade 3 or 4, which is similar to the situation observed in phase 1 studies (Palanca-Wessels et al., 2015) but do not lead to discontinuation of treatment in polatuzumab vedotin-R (Pola-R) patients (Morschhauser et al., 2019). However, severe neutropenia can occur in less than 10% of patients when B and polatuzumab vedotin are combined, which can result in treatment interruption. Therefore, it is necessary to conduct a close follow-up of patients who receive polatuzumab vedotin-BR (Pola-BR) (Lee et al., 2012). This study revealed that the Pola-BR group had a higher prevalence of grade 3 or greater leukopenia, but BR had a similar incidence of febrile neutropenia. The incidence of anemia and thrombocytopenia at all levels was higher in the Pola-BR group than in the BR group. Therefore, preventive treatment and monitoring must be provided by physicians prior to using polatuzumab vedotin to resolve AEs in most patients (Assi et al., 2021). To illustrate, granulocyte colony-stimulating factor and other preventive drugs can be utilized to treat neutropenia (Deeks, 2019).Additionally, we observed a significant increase in the signal intensity of AEs related to myelosuppression in our analysis, which is consistent with the signal intensity reported in the instructions and clinical safety data. Regular monitoring of blood and bone marrow examinations is necessary to prevent fatal AEs caused by myelosuppression, a common AE of anti-tumor drugs.

It’s noteworthy that polatuzumab vedotin is linked to infections and infestations, which include COVID-19, pneumonia, cytomegalovirus infection, and sepsis. Cancer patients are thought to be particularly vulnerable to novel coronavirus infections and more severe COVID-19 symptoms. Systemic immunosuppression may be responsible for this, both directly because of tumor growth and indirectly because of the effects of anti-cancer treatment (Liu et al., 2020). Cancer patients with COVID-19 may also experience various complications (Huang et al., 2020; Liang et al., 2020; Xu et al., 2020). Acute respiratory distress syndrome (28.6%), pulmonary embolism (7.1%), septic shock (3.6%), and acute myocardial infarction (3.6%) are the most common complications and causes of death among these patients. In a phase 1b/2 study of mosunetuzumab combined with polatuzumab vedotin for DLBCL, 3.3% of patients were diagnosed with severe COVID-19. COVID-19 had no specific antiviral treatment, and the treatment given or under study included non-invasive or invasive mechanical ventilation to prevent respiratory disorders (Ñamendys-Silva, 2020) drugs including corticosteroids (such as dexamethasone) (Ledford, 2020) antimalarial drugs (such as hydroxychloroquine) (Arshad et al., 2020) and antiviral drugs (such as lopinavir or ritonavir) (Rosa and Santos, 2020) Recovery plasma therapy (Piechotta et al., 2020) and anti-inflammatory antibodies (such as anti IL-6 receptor antibody tocilizumab) (Michot et al., 2020). The impact of pneumonia on cancer populations is particularly severe, with a higher incidence rate and mortality rate than any other infectious complications (Evans and Ost, 2015). In the 1b/2 phase GO29365 study, pneumonia accounted for 16% of grade 3 AEs in R/R DLBCL patients treated with polatuzumab vedotin. In order to prevent any more related infections, it is essential to receive appropriate antibiotic treatment and supportive care (Wang et al., 2022). Cytomegalovirus infection, which is a genetic product of human cytomegalovirus, is found in a variety of human malignant tumors and is typically connected to tumor cells and the tumor vascular system (Cobbs, 2019). Saburi et al. (2023) conducted research which revealed that 19.2% of R/R DLBCL patients with MYC translocation were diagnosed with cytomegalovirus reactivation when they received Pola-BR. Prophylactic treatment with acyclovir resulted in the absence of symptomatic diseases caused by cytomegalovirus in patients with cytomegalovirus reactivation. Patients who have solid tumors or hematological tumors are more prone to sepsis, and it is closely linked to the use of anti-tumor drugs, especially ADC type anti-tumor drugs (Xia et al., 2022). Cancer patients are at a higher risk of developing sepsis than the general population because of the suppression of inflammation and immune responses. The host’s immune response can be altered by cancer treatment, which can lead to increased susceptibility to infection (Williams et al., 2023). The risk of patient death can be increased by the rapid progression of sepsis into septic shock and multiple organ failure (Cecconi et al., 2018). Some patients in the Pola-BR group stopped treatment due to sepsis in a clinical study on its safety and effectiveness (Sehn et al., 2020). Therefore, clinical vigilance and immediate treatment are essential for managing sepsis patients (Huang et al., 2019).

It's worth noting that there is a discrepancy in reporting nervous system disorders, such as peripheral neuropathy, anxiety, peripheral sensory neuropathy, and so on. The occurrence may be caused by the action of unbound MMAE in the circulation, which may also be the cause of reducing or stopping treatment. Grade 1–2 peripheral neuritis was experienced by 27% of NHL patients when polatuzumab vedotin was administered as a single drug at 2.4 mg/kg, while grade 3–4 peripheral neuritis was experienced by 9%. The peripheral neuropathy that has been observed is mainly sensory and rarely encompasses motor events (Palanca-Wessels et al., 2015). Peripheral neuropathy patients experience a delay in dose before resuming treatment at a reduced dose until the neuropathy subsides. However, further investigation is needed to investigate the comprehensive impact of these measures on the reversibility of peripheral neuropathy (Palanca-Wessels et al., 2015) The Phase II study of polatuzumab vedotin combined with rituximab proved that neuropathy occurred in 40%, but only grade 1–2 toxicity was mentioned (Morschhauser et al., 2019). The cumulative risk of peripheral neuropathy may be higher in patients who have previously received treatment with Vinca alkaloids in these studies. To determine the correlation between conjugate exposure and treatment duration and the risk of peripheral neuropathy, Lu et al. (2017) developed an event time model using data from phase I and II studies, which supports limiting the dose of polatuzumab vedotin in combination with chemotherapy or pathway inhibitors to 1.8 mg/kg (Pfeifer et al., 2015). In the randomized GO29365 study (Sehn et al., 2020), when the polatuzumab vedotin dose was 1.8 mg/kg, 43.6% of patients in the Pola-BR group developed peripheral neuropathy. The reported events were limited to Grade 1 and Grade 2, and most of the symptoms were relieved after treatment.

Unexpected and significant safety signals, such as immune system disorders, cardiac disorders, renal and urinary disorders, skin and subcutaneous tissue disorders, vascular disorders, were detected in our analysis. However, these AEs are not explicitly mentioned in the polatuzumab vedotin manual. The PT signals involved, in order of the number of cases, are cardiac failure, hypogammaglobulinaemia, femoral neck fracture, erythema multiform, thrombophlebitis, azotaemia. The newly emerging signals, particularly cardiac failure and hypogammaglobulinaemia, need to be given a great deal of attention. Cardiotoxicity is accompanied by a history of anti-tumor drug treatment, and whether it is traditional chemotherapy (Cadeddu Dessalvi et al., 2021), new targeted therapies (Tajiri et al., 2017) or immunotherapy (Knobloch et al., 2008), it may lead to cardiac related AEs. Myocardial infarction was observed in a clinical study of polatuzumab vedotin combined with immunochemotherapy for previously untreated DLBCL patients (Tilly et al., 2022). It is crucial to identify patients who are at risk of developing heart failure who are being treated with this treatment and it is recommended that cancer patients with cardiac risk factors undergo cardiac monitoring, which includes LVEF assessment (Ewer et al., 2021). The clinical manifestation of hypogammaglobulinemia is recurrent infections, with chronic upper and lower respiratory diseases being the most common. Reducing the severity of chronic upper and lower respiratory diseases, improving lung function and reaching normal IgG levels, and using immunoglobulin replacement therapy are currently acceptable treatment targets for patients with hypogammaglobulinemia (Long et al., 1987). To enhance the prognosis of the aforementioned AEs, early diagnosis, appropriate treatment, and prevention are considered important measures.

We conducted a subgroup analysis on gender to examine possible differences in the incidence of AE between populations of different genders. It was observed that male patients reported more PTs linked to polatuzumab vedotin than female patients, and the number of reported cases was higher than that for female patients. The reason may be that the incidence rate of DLBCL in men is higher than that of DLBCL in women (Wang, 2023). Male patients who use polatuzumab vedotin to some extent exceed female patients, and male patients report more AEs. This is consistent with our research findings on the reporting ratio of AEs. The male population made up 49% of the total reported cases among the 1,521 patients reported in Table 1, which is the reason for our hypothesis. Furthermore, the population that lacks gender is responsible for 14.7% of the total reported cases. Based on this, it is necessary to further track and report the occurrence of AEs in female patients after using this medication.

We further conducted an age-based subgroup analysis to examine whether age is a factor in the occurrence of polatuzumab vedotin-related AEs. The findings reveal that elderly people (≥65 years old) have a higher frequency of AEs. Compared to patients between 18 and 65, there has been a significant increase in the number of AE cases. The top three PT with ROR values detected in the two groups of people are not the same. The strongest signals detected in the population aged 65 and above are cytomegalovirus test positive, cytomegalovirus enterocolitis, and cytomegalovirus infection reactivation. The strongest signals detected in the population aged 65 and below are cytomegalovirus test positive, absolute lymphadenopathy, and hypogammaglobulinaemia. Therefore, there are certain differences in the occurrence of AEs among different age groups. The majority of AEs in elderly patients are due to infection, which may be related to the increased risk of severe infection (Thandra et al., 2021). Due to the fact that elderly patients often have complex/multiple comorbidities, doctors may need to be more cautious in their treatment of these patients. A phase 3 trial is currently being conducted to investigate the age adaptive combination of Pola-R-CHP and dose attenuated chemotherapy in the elderly patient population (ClinicalTrials.gov number, NCT04332822). In addition, Malecek et al. (2021) found that in young patients and patients without severe comorbidities, Pola-BR has the potential to serve as a bridge drug in more intensive treatment, with a high level of efficacy and tolerability.

To investigate the occurrence of AEs that are related to death cases, we conducted a subgroup analysis of cases where polatuzumab vedotin-induced AEs led to death. High-risk AEs in fatal cases include blood lactate dehydrogenase increased, cytopenia, and hydronephrosis. Dimou et al. (2021) studied that in the treatment of invasive B-cell lymphoma with Pola-BR combination therapy, 55% of patients experienced ≥ grade 3 AEs, mainly hematological AEs. Treatment interruption and death during treatment were mainly due to disease progression. These manifestations are frequently connected to the primary disease, or with disease progression or recurrence during the treatment process (Song et al., 2023). During treatment with polatuzumab vedotin, patients should receive close monitoring for the aforementioned AEs. In this research, it was discovered that PT signals that were not mentioned in the drug instructions were manifested as blood lactate dehydrogenase increased, hydronephrosis and hypogammaglobulinaemia. Furthermore, blood lactate dehydrogenase increased and hydronephrosishave a higher risk of mortality. This signal may be a new and important signal. When using drugs, it is necessary to strengthen the monitoring of biochemical indicators, kidneys, and gamma globulin to prevent the occurrence of malignant AEs that threaten patient safety.

In order to assess the time of occurrence of AEs to polatuzumab vedotin, we carried out an analysis on the time of occurrence of AEs to polatuzumab vedotin. The results indicated that the median time for detecting AEs with polatuzumab vedotin was 18.5 (5∼57.75) days, and most AEs occurred within 162 days, but AEs may still occur within 1 year (Walji and Assouline, 2020). As per these results, it is recommended that we pay special attention to AEs occurring in patients within the first month. Patient pain can be eased by the early identification of AEs caused by polatuzumab vedotin treatment (Lin et al., 2024), and longer follow-up periods will be required in future clinical studies to monitor AEs related to polatuzumab vedotin (Gouni et al., 2022).

### Limitations

However, although data mining techniques have advantages in analysing clinical safety issues in real-world, there were also certain limitations. The FAERS database, as a voluntary, passive, and non-mandatory reporting system, faces inherent challenges. These include incompleteness, inaccuracy, inconsistency, and delay in reporting AEs. These limitations stem from various factors, primarily the lack of detailed patient characteristics, drug exposure information, and outcome details, such as the dose and duration of polatuzumab vedotin use, as well as whether patients received other treatment regimens and the sequence of medication. These factors may influence the associations observed and the study outcomes. Therefore, it is essential to carefully consider these limitations, particularly when interpreting the research results. To overcome these limitations and provide more robust insights, further prospective clinical studies are urgently needed. Additionally, the mechanisms underlying the association between polatuzumab vedotin use and AEs remain unclear, underscoring the need for fundamental research to address these uncertainties and advance our understanding of ADC drug targeted therapy. Finally, all signal detection results can only suggest that there is a statistical correlation; whether there is a real causal relationship still needs further evaluation and research.

## Conclusion

Based on FAERS database, our study investigated and analyzed AE signals associated with polatuzumab vedotin to enhance clinical medication safety. The AEs identified in this study largely align with those documented in the drug’s manual. Additionally, we uncovered AEs that were not previously listed, including cardiac failure, hypogammaglobulinaemia, femoral neck fracture, erythema multiform, etc. These findings supplement the limited sample size of pre-market clinical studies. However, we have not yet explored the correlation between polatuzumab vedotin-related AEs and the drug itself, underscoring the need to augment safety information. Further research is still warranted to elucidate the relationship between novel polatuzumab vedotin-related AEs and the drug and to refine polatuzumab vedotin’s safety profile. Comprehensive understanding of polatuzumab vedotin AEs is crucial for mitigating usage risks, promptly identifying AEs, and appropriately managing them to optimize patient treatment outcomes.

## Data Availability

The original contributions presented in the study are included in the article/Supplementary Material, further inquiries can be directed to the corresponding authors.
