# Supplementary material for: A real-world pharmacovigilance study of polatuzumab vedotin based on the FDA adverse event reporting system (FAERS)
Source: Front Pharmacol. 2024 Jun 25;15:1405023. doi: 10.3389/fphar.2024.1405023 (PMC11231375; doi:10.3389/fphar.2024.1405023)
Supplement: Supplementary file 3 [file Table1.DOCX]

Supplementary Table 1 Four main algorithms for evaluating the potential association between polatuzumab and AEs.

| Method | Formula | Threshold |
| --- | --- | --- |
| ROR | $ROR=\frac{a/c}{b/d}$  $ROR_{95\%CI}=e^{\ln(ROR)\pm1.96\sqrt{(\frac{1}{a}+\frac{1}{b}+\frac{1}{c}+\frac{1}{d}})}$ | $a\geq3,ROR_{95\%CIlower limit}>1$ |
| PRR | $PRR=\frac{a/(a+b)}{c/(c+d)}$  $E=\frac{(a+b)(a+c)}{(a+b+c+d)}$  $\chi^{2}=\sum\frac{(a-E-0.5)^{2}}{E}$ | $a\geq3,PRR\geq2,\chi^{2}\geq4$ |
| BCPNN | $IC={log}_{2}\frac{a+0.5}{a_{exp}+0.5}$  $a_{exp}=\frac{\left( a+b \right)*(a+c)}{(a+b+c+d)}$  ${IC}_{025}=IC-3.3*\left( a+0.5 \right)^{-0.5}-2*{(a+0.5)}^{-1.5}$ | $a\geq3,IC_{025}>0$ |
| MGPS | $EBGM=\frac{a*(a+b+c+d)}{\left( a+c \right)*(a+b)}$  ${EBGM}_{05}=e^{ln(EBGM)}-1.64*{(\frac{1}{a}+\frac{1}{b}+\frac{1}{c}+\frac{1}{d})}^{-0.5}$ | $a\geq3, {EBGM}_{05}\geq2$ |

Note: α. Number of reports containing both the target drug and adverse reactions to the target drug; b. Number of reports of adverse reactions to other drugs that contain the target drug; c. Number of reports of adverse reactions to the target drug that contain other drugs; d. Number of reports of adverse reactions to other drugs that contain other drugs and other drugs. ROR, reporting odds ratio; ROR_95%CI_, 95% confidence interval of the ROR; PRR, Proportional Reporting Ratio; χ^2^, chi-squared; IC, information component; IC_025_, the lower limit of the 95% confidence interval of the IC; EBGM, empirical Bayesian geometric mean; EBGM_05_, empirical Bayesian geometric mean lower 95% confidence interval for the posterior distribution.
